# Safety and Pharmacokinetics of Taniborbactam (VNRX-5133) with Cefepime in Subjects with Various Degrees of Renal Impairment

**DOI:** 10.1128/aac.00253-22

**Published:** 2022-08-03

**Authors:** James A. Dowell, Thomas C. Marbury, William B. Smith, Tim Henkel

**Affiliations:** a Pharmacology Development Services, LLC, Collegeville, Pennsylvania, USA; b Orlando Clinical Research Center, Orlando, Florida, USA; c Alliance for Multispecialty Research, University of Tennessee Medical Center, Knoxville, Tennessee, USA; d Venatorx Pharmaceuticals, Inc., Malvern, Pennsylvania, USA

**Keywords:** taniborbactam, cefepime, cefepime-taniborbactam, VNRX-5133, beta-lactamase inhibitor, drug safety, pharmacokinetics, renal impairment

## Abstract

Taniborbactam, an investigational β-lactamase inhibitor that is active against both serine- and metallo-β-lactamases, is being developed in combination with cefepime to treat serious infections caused by multidrug-resistant Gram-negative bacteria. Anticipating the use of cefepime-taniborbactam in patients with impaired renal function, an open-label, single-dose clinical study was performed to examine the pharmacokinetics of both drugs in subjects with various degrees of renal function. Hemodialysis-dependent subjects were also studied to examine the amounts of cefepime and taniborbactam dialyzed. Single intravenous infusions of 2 g cefepime and 0.5 g taniborbactam coadministered over 2 h were examined, with hemodialysis-dependent subjects receiving doses both on- and off-dialysis. No subjects experienced serious adverse events or discontinued treatment due to adverse events. The majority of adverse events observed were mild in severity, and there were no trends in the safety of cefepime-taniborbactam related to declining renal function or the timing of hemodialysis. Clinically significant and similar decreases in drug clearance with declining renal function were observed for both cefepime and taniborbactam. The respective decreases in geometric mean clearance for subjects with mild, moderate, and severe renal impairment compared to subjects with normal renal function were 18%, 63%, and 78% for cefepime and 15%, 63%, and 81% for taniborbactam, respectively. Decreases in clearance were similar for both drugs and were shown to be proportional to decreases in renal function. Both cefepime and taniborbactam were dialyzable, with similar amounts removed during 4 h of hemodialysis. This study is registered at ClinicalTrials.gov as NCT03690362.

## INTRODUCTION

Taniborbactam (formerly VNRX-5133) is a broad spectrum, cyclic boronate β-lactamase inhibitor that is being developed with cefepime as a partnered antibiotic. Taniborbactam exhibits potent competitive inhibition of the Ambler Class A, C, and D serine-β-lactamases, and the Ambler class B metallo-β-lactamases, such as the Verona integron-encoded metallo-β-lactamases (VIM) and the New Delhi metallo-β-lactamases (NDM) (1–4). Cefepime is a fourth-generation cephalosporin antibiotic with extended spectrum activity against Gram-positive and Gram-negative bacteria. In the United States, cefepime is approved for the treatment of moderate to severe pneumonia, uncomplicated and complicated urinary tract infections, including pyelonephritis, uncomplicated skin and skin structure infections, and complicated intra-abdominal infections that are caused by susceptible strains of designated microorganisms as well as for empirical therapy for febrile neutropenia (5). The combination of cefepime and taniborbactam (cefepime-taniborbactam) is being investigated as a treatment for complicated urinary tract infections (NCT03840148) and for other serious infections in which multidrug resistant Gram-negative pathogens occur, such as hospital-acquired and ventilator-associated pneumonia (6, 7). In nonclinical models of infection, taniborbactam has been shown to potentiate cefepime activity against Gram-negative pathogens with β-lactamase-mediated resistance (8–12).

As an antibiotic combination that will be used to treat life-threatening infections, cefepime-taniborbactam is likely to be administered to patients with various degrees of renal impairment. In clinical studies, following the administration of 0.25 to 2 g cefepime, at least 80% of the cefepime dose was excreted unchanged in urine (13–15). In patients with renal impairment, the cefepime terminal elimination half-life (*t*_1/2_) significantly increases up to 6-fold in patients with creatinine clearance (CL_CR_) values of <10 mL/min, and dosing adjustments are required for patients with various degrees of renal impairment (5, 13, 16). The pharmacokinetics of taniborbactam in healthy volunteers are generally similar to that of cefepime (17). Clinical studies have shown that taniborbactam is primarily eliminated unchanged in urine and at steady-state; approximately 89% of the taniborbactam dose is recovered in urine as unchanged parent compound (18).

The objective of this study was to evaluate the safety and pharmacokinetics of cefepime-taniborbactam in subjects with mild, moderate, or severe renal impairment as well as in subjects with end-stage renal disease (ESRD) requiring hemodialysis.

## RESULTS

### Subjects.

A total of 33 subjects were enrolled in the study, received the combination treatment of single intravenous infusions of 2 g cefepime and 0.5 g taniborbactam, and had pharmacokinetics assessed. A summary of demographics and baseline estimates of renal function for subjects in each group can be found in Table 1. Subject sex, age, and weight were similar across the nondialysis groups. For subjects in the Normal group, the estimated creatinine clearance (eCL_CR_) determined by the Cockcroft-Gault equation (19) ranged from 100.7 to 174.0 mL/min. For subjects in the Mild, Moderate, and Severe groups, enrollment was based on estimated glomerular filtration rate (eGFR) as determined by the Modification of Diet in Renal Disease (MDRD) equation (20, 21). Across these renal impairment groups, subject eGFR values ranged between 5.5 and 85.0 mL/min/1.73m^2^.

**TABLE 1:** Subject demographics and baseline renal function^*a*^

	Renal group				
Variable	Normal (*n* = 8)	Mild (*n* = 6)	Moderate (*n* = 6)	Severe (*n* = 6)	Dialysis (*n* = 7)
Age, yr, mean (SD)	49 (11)	58 (7)	62 (11)	60 (12)	48 (11)
Sex, n (%)					
Male	6 (75.0)	3 (50.0)	3 (50.0)	3 (50.0)	7 (100.0)
Female	2 (25.0)	3 (50.0)	3 (50.0)	3 (50.0)	0 (0.0)
Wt, kg, mean (SD)	87.0 (13.7)	84.6 (15.3)	81.4 (20.1)	78.3 (20.2)	87.5 (12.7)
BMI, kg/m^2^, mean (SD)	30.5 (4.7)	30.1 (3.1)	28.2 (3.9)	28.9 (5.1)	27.9 (4.1)
Race, n (%)					
White	4 (50.0)	5 (83.3)	5 (83.3)	5 (83.3)	0 (0.0)
Black or African American	3 (37.5)	1 (16.7)	1 (16.7)	1 (16.7)	7 (100.0)
Other	1 (12.5)	0 (0.0)	0 (0.0)	0 (0.0)	0 (0.0)
Renal Function, mean (SD)					
eCL_CR_, mL/min	131.0 (25.8)	94.9 (13.3)	47.1 (13.7)	32.6 (19.7)	10.3 (3.5)
eGFR, mL/min/1.73m^2^	102.7 (13.6)	74.6 (7.9)	36.1 (5.2)	22.5 (9.4)	6.1 (1.3)

a eCL_CR_ = estimated creatinine clearance determined by Cockcroft-Gault; eGFR = estimated GFR determined by MDRD equation.

Subjects in the Dialysis group (*n* = 7) were all hemodialysis-dependent, male, and 27 to 56 years of age. This group had a mean (SD) weight of 87.5 (12.7) kg.

### Safety.

Single doses of 2 g cefepime and 0.5 g taniborbactam, administered in combination as a 2 h intravenous infusion, were found to be safe and well-tolerated in otherwise healthy subjects with various degrees of renal impairment and in otherwise healthy subjects that were hemodialysis-dependent. There were no deaths reported in the study. There were no apparent trends in the incidence, type, or severity of treatment-emergent adverse events with declining renal function or timing of hemodialysis. A total of 7 subjects experienced 8 treatment emergent adverse events; these events were migraine, headache, diarrhea, abdominal pain upper, pain in jaw, drug withdrawal syndrome (caffeine withdrawal), and *Clostrioides difficile* infection. The majority of adverse events were mild in severity (7 of 8) and considered related to treatment (7 of 8). There were no hematology, chemistry, urinalysis, or coagulation adverse events reported. The majority of postbaseline laboratory and vital sign abnormalities were mild, and there were no apparent trends observed in frequency or severity with declining levels of renal function or with the timing of hemodialysis. There were no treatment-emergent adverse events related to ECG or vital signs. There was no evidence of renal toxicity based on the monitoring of blood urea nitrogen, serum creatinine, eGFR, and microscopic evaluation of urine for renal tubular epithelial casts.

### Pharmacokinetics.

**FIG 1:**
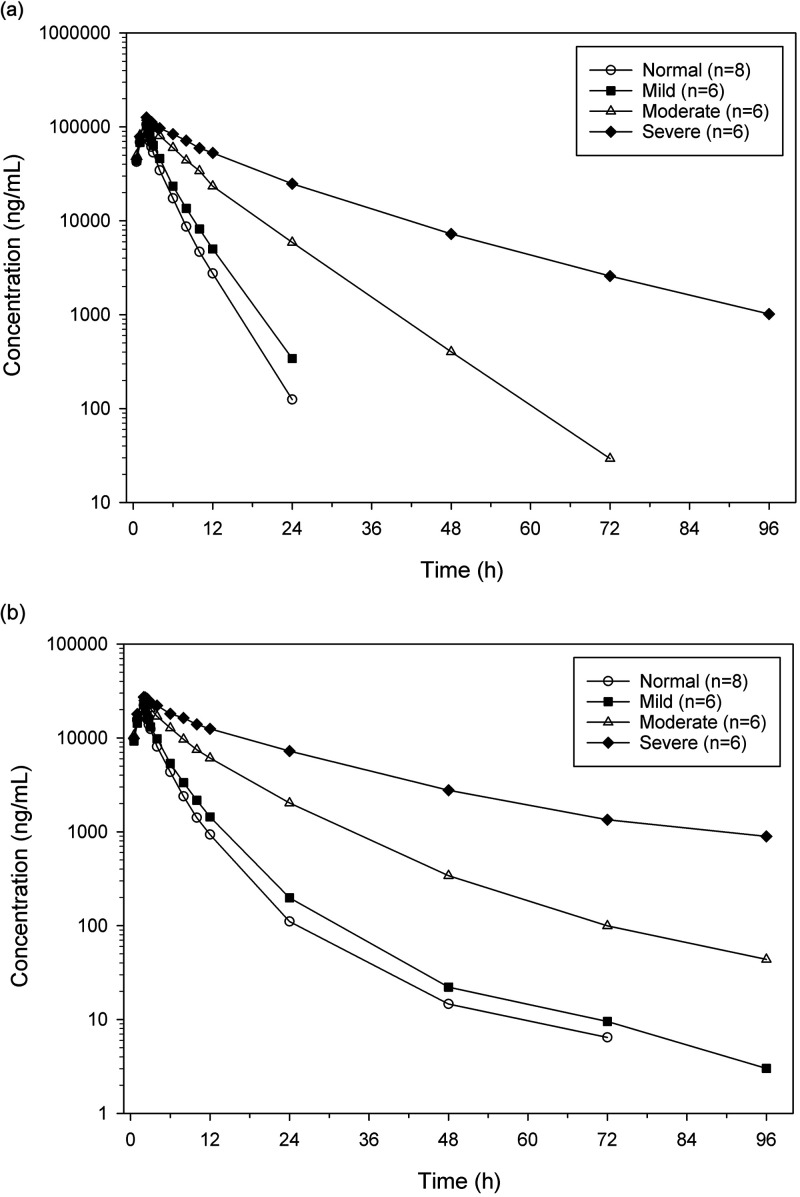
Mean cefepime and taniborbactam plasma concentrations across renal groups (nondialysis). (a) Cefepime, logarithmic concentration scale. (b) Taniborbactam, logarithmic concentration scale.

Mean cefepime and taniborbactam plasma concentration-time profiles are compared for the nondialysis renal groups in Fig. 1. Summaries of the cefepime and taniborbactam pharmacokinetic parameters for each of the nondialysis renal groups are shown in Table 2.

**TABLE 2:** Summary of pharmacokinetic parameters by renal group (nondialysis groups)^*a*^

		Renal impairment		
Parameter, unit^*b*^	Normal (*n* = 8)	Mild (*n* = 6)	Moderate (*n* = 6)	Severe (*n* = 6)
Cefepime^*c*^				
*Cmax*, μg/mL	102 (25.3)	101 (19.4)	124 (20.6)	129 (21.8)
AUC_inf_, h·μg/mL	343 (13.2)	418 (9.0)	913 (20.0)	1589 (69.0)
*t*_1/2_, h	2.53 (0.53)	3.03 (0.39)	5.53 (1.34)	10.12 (5.16)
*V*_Z_, L	20.2 (20.5)	20.0 (21.2)	16.3 (25.6)	16.4 (21.1)
CL, L/h	5.65 (13.8)	4.61 (11.6)	2.09 (23.2)	1.23 (66.6)
Taniborbactam				
*Cmax*, μg/mL	22.0 (11.2)	22.8 (22.6)	26.9 (24.1)	27.9 (21.7)
AUC_inf_, h·μg/mL	83.6 (11.4)	97.4 (11.5)	225 (22.5)	445 (79.3)
*t*_1/2_, h	10.2 (2.6)	19.5 (9.9)	17.6 (2.6)	21.3 (10.1)
*V*_Z_, L	82.0 (33.4)	123.0 (59.5)	53.1 (35.6)	31.5 (43.5)
CL, L/h	5.79 (11.7)	4.95 (13.9)	2.12 (25.8)	1.10 (76.7)
CL_R_, L/h	4.37 (17.8)^*d*^	4.23 (22.7)	1.59 (21.0)	0.76 (120.3)

a *Cmax* = maximum plasma concentration; AUC_inf_ = area under the plasma concentration versus time curve, extrapolated through infinity; *t*_1/2_ = terminal elimination half-life; *V*_z_ = volume of distribution estimated using the terminal phase; CL = total body clearance; CL_R_ = renal clearance.

b Geometric mean (geometric coefficient of variation [%]) shown for all parameters except for *t*_1/2_, which shows the mean (standard deviation).

c Cefepime was not assayed in urine, and cefepime CL_R_ was not estimated in study.

d *n* = 7, as a subject was excluded from the summary statistics because of a missed urine collection.

Statistical comparisons of maximum observed concentration (*Cmax*), area under the concentration versus time curve (AUC), systemic clearance (CL), and volume of distribution based on the terminal elimination phase (*V*_Z_) for each of the renal impairment groups to the Normal group were performed for cefepime and taniborbactam. Least-square geometric mean ratios (GMRs; Renal Impairment Group/Normal [%]) and their 90% confidence intervals (CIs) were calculated. For the Mild/Normal ratio of cefepime and taniborbactam pharmacokinetic parameters, all 90% CIs contained 100% except for taniborbactam *V*_Z_, which had a GMR (90% CI) of 149.94% (102.40%, 219.57%). For cefepime and taniborbactam, a statistically significant increase in AUC, extrapolated through infinity (AUC_inf_), was observed for all nondialysis-dependent renal impairment groups compared with Normal, as assessed using the 90% CIs and the significance boundaries of 80.00% to 125.00%. Inversely, cefepime and taniborbactam CL were significantly decreased in these groups compared with Normal. Similar trends of increased exposure and decreased CL as a function of decreasing renal function were observed for cefepime and taniborbactam (Table 2).

**FIG 2:**
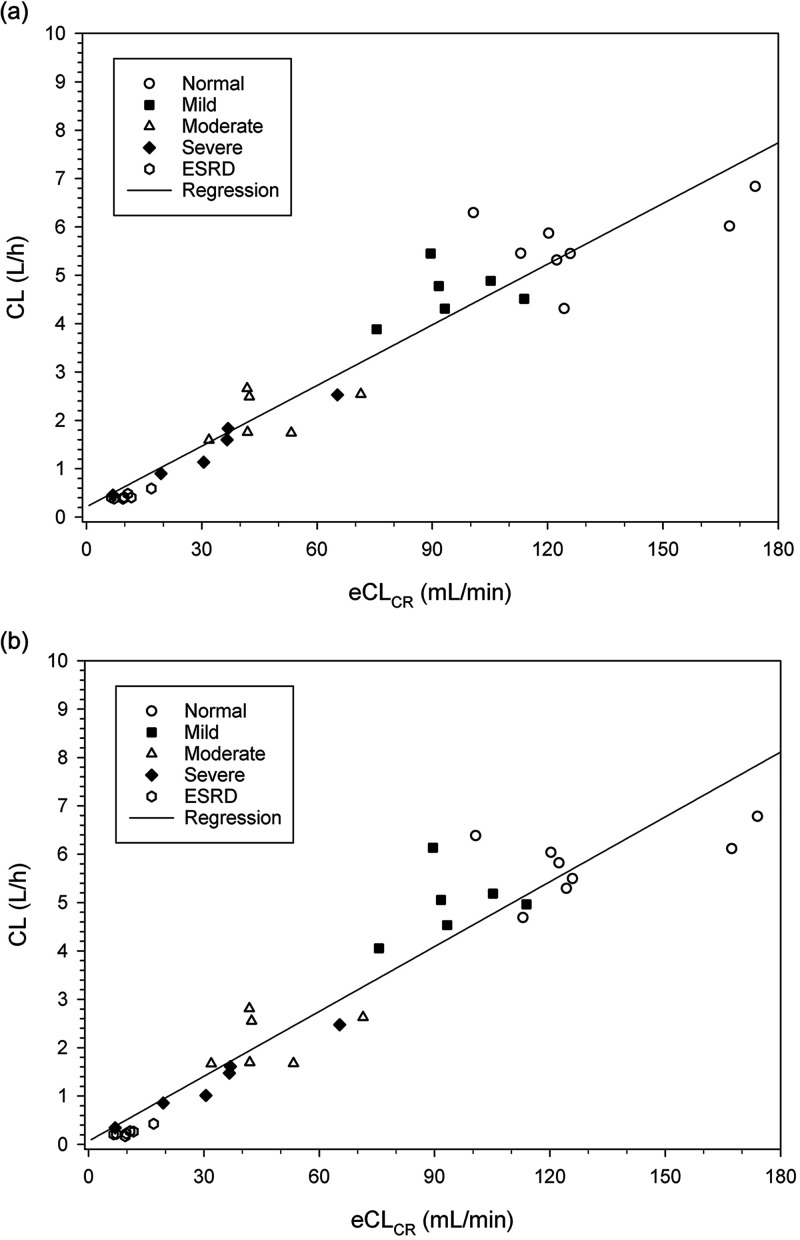
Cefepime and taniborbactam CL versus eCL_CR_, showing linear regression. (a) Cefepime. (b) Taniborbactam.

Significant trends in the individual pharmacokinetic parameters as a function of individual measures of renal function were observed for both cefepime and taniborbactam. A robust trend was observed for individual CL as a function of individual eCL_CR_ using linear regression, for both cefepime and taniborbactam (Fig. 2). The data set for these regressions was supplemented with ESRD subjects from the Dialysis group, using data from the Off-dialysis treatment period. This was done to include more subjects with lower degrees of renal function (eGFR_MDRD_ <15 mL/min/1.73 m^2^). The relationships between drug CL and eCL_CR_ were well-defined by linear regression; the R^2^ values were 0.9130 and 0.9054 for cefepime and taniborbactam, respectively. The CL values and changes in CL values were similar for cefepime and taniborbactam. The estimated slopes for the relationship of drug CL as a function of eCL_CR_ were 0.0418 and 0.0447 for cefepime and taniborbactam, respectively.

A total of 7 subjects were enrolled in the Dialysis group and received taniborbactam and cefepime in both the On-dialysis and Off-dialysis treatment periods. Hemodialysis was performed in one of the treatment periods (Period 1, On-dialysis) with hemodialysis durations of between 4 and 4.13 h across all subjects. One subject in the Dialysis group was replaced in the study because hemodialysis during the On-dialysis treatment period started almost 8 h after the start of infusion (SOI), compared with the protocol-specified 4 h post-SOI. Pharmacokinetic data were still collected for this subject during both treatment periods, which allowed for a limited assessment of the effect of hemodialysis timing. No obvious differences were noted for this subject, compared with the other Dialysis subjects, as to the amount of cefepime or taniborbactam dialyzed. This subject’s On-dialysis period was excluded from all pharmacokinetic parameter summary statistics and statistical comparisons but was used in the calculation of the dialysis parameters.

**FIG 3:**
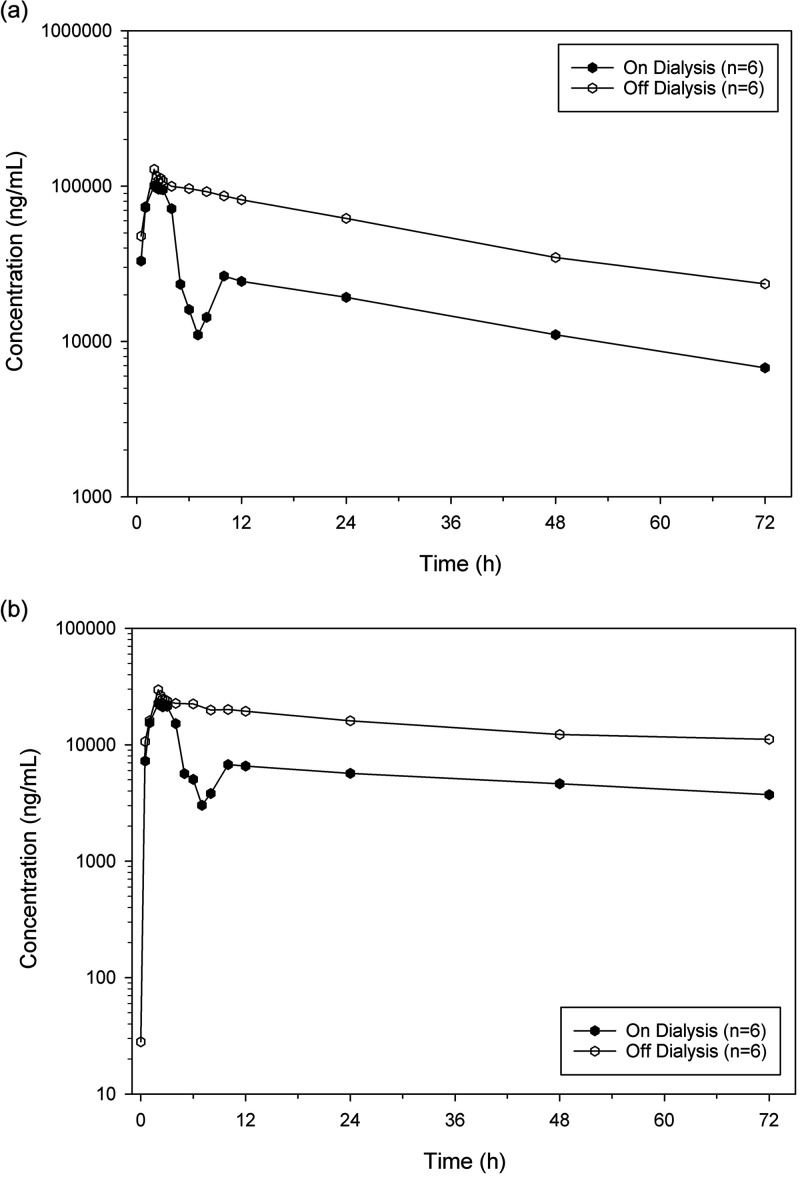
Comparison of mean cefepime and taniborbactam plasma concentrations in the on-dialysis and off-dialysis treatment periods (dialysis subjects). (a) Cefepime, logarithmic concentration scale. (b) Taniborbactam, logarithmic concentration scale.

For the Dialysis subjects, the mean cefepime and taniborbactam plasma concentration-time profiles from the On-dialysis and Off-dialysis treatment periods are compared in Fig. 3. Summaries of the cefepime and taniborbactam pharmacokinetic parameters comparing the On-dialysis and Off-dialysis periods are shown in Table 3. All subjects were completely or near completely anuric, and there were no urine-associated pharmacokinetic parameters summarized for Dialysis subjects.

**TABLE 3:** Comparison of pharmacokinetic parameters in the on-dialysis and off-dialysis treatment periods (dialysis group)^*a*^

	Hemodialysis	
Parameter, unit^*b*^	On-dialysis (*n* = 6)^*c*^	Off-dialysis (*n* = 7)
Cefepime		
*Cmax*, μg/mL	105 (13.0)	165 (77.2)
AUC_inf_, h·μg/mL	1597 (16.7)	4549 (17.7)
*t*_1/2_, h	32.0 (4.3)	29.7 (5.8)
*V*_Z_, L	56.5 (9.0)	18.2 (8.8)
CL, L/h	1.23 (17.6)	0.432 (15.9)
Taniborbactam		
*Cmax*, μg/mL	23.4 (9.5)	37.7 (73.7)
AUC_inf_, h·μg/mL	851 (23.0)	2003 (32.1)
*t*_1/2_, h	83.7 (21.5)	70.8 (22.3)
*V*_Z_, L	68.1 (15.1)	23.7 (13.4)
CL, L/h	0.579 (23.0)	0.245 (29.8)

a *Cmax* = maximum plasma concentration; AUC_inf_ = area under the plasma concentration versus time curve, extrapolated through infinity; *t*_1/2_ = terminal elimination half-life; *V*_z_ = volume of distribution estimated using the terminal phase; CL = total body clearance.

b Geometric mean (geometric coefficient of variation [%]) shown for all parameters except for *t*_1/2_, which shows the mean (standard deviation).

c One subject excluded in the summary statistics because dialysis occurred 7.8 h after the start of drug infusion.

Hemodialysis was shown to substantially remove cefepime and taniborbactam from plasma at similar rates. Geometric mean cefepime and taniborbactam AUCs were decreased following hemodialysis by 65% and 58%, respectively. Mean cefepime and taniborbactam *Cmax* values were comparable between Off-dialysis and On-dialysis treatments when anomalous values from a single subject (thought to be due to a sampling error) were excluded. The mean cefepime and taniborbactam *t*_1/2_ remained relatively unaffected by hemodialysis.

Statistical comparisons of treatment periods within the Dialysis group were performed for cefepime and taniborbactam *Cmax*, AUC_inf_, CL, and *V*_Z_, calculating the least-squares GMR (On-dialysis/Off-dialysis [%]) and their respective 90% CIs. Significant changes were noted in all assessed pharmacokinetic parameters. The GMRs (90% CIs) for cefepime and taniborbactam AUC_inf_ were 33.46% (30.39%, 36.83%) and 39.29% (33.20%, 46.48%), respectively.

The estimated drug-dependent dialysis parameters were similar for both drugs. The mean (SD) dialysis clearances (CL_d_) were 117 (12.3) mL/min and 102 (5.3) mL/min for cefepime and taniborbactam, respectively. The mean (SD) cefepime and taniborbactam hemodialysis extraction ratios (HER) were 47.4% (7.7%) and 49.7% (7.1%), respectively. 2 of the 7 subjects had On-dialysis venous samples collected from the contralateral arm rather than from the dialysis output, and CL_d_ could not be calculated for these subjects.

## DISCUSSION

Cefepime and taniborbactam are both primarily excreted unchanged in urine, and the pharmacokinetics of both drugs are similarly impacted by renal impairment. Generally, the distributions of drug clearance and exposure for both drugs were similar and overlapping for the Normal and Mild groups, indicating only a small effect due to renal impairment for subjects with an eGFR value of >60 mL/min/1.73m^2^. Continued decreases in renal function led to significant decreases in CL and increases in exposure for both drugs. For subjects in the Moderate group (eGFR = 30 to 59 mL/min/1.73 m^2^), geometric mean CL decreased 63% for both drugs. In the Severe group (eGFR <30 mL/min/1.73 m^2^), geometric mean CL decreases of 78% and 81% were observed for cefepime and taniborbactam, respectively. AUC_inf_ increased across groups in an inverse manner. Modest decreases in distributive drug volumes were observed for both drugs with decreasing renal function, with similarly modest and associated increases in *Cmax*. The mean terminal half-lives (t_1/2_) of cefepime and taniborbactam in the Normal group were 2.53 h and 10.2 h, respectively, and t_1/2_ increased with decreasing renal function. The longer taniborbactam t_1/2_ is due to a longer terminal phase in the drug’s biphasic elimination, which describes only a small fraction of the drug’s overall exposure (Fig. 1).

In the presented study, subjects with normal renal function were enrolled based on eCL_CR_, and eGFR was used for subjects with renal insufficiency. This was based on regulatory guidance at the time of the study (22). The use of eGFR to quantitate renal insufficiency, typically using the MDRD equation, has become an accepted practice in these types of otherwise healthy volunteer studies. In this study, the enrollment criteria produced a good range and distribution of renally insufficient subjects that allowed for a robust assessment of the relationship between drug pharmacokinetics and impaired renal function, regardless of which serum creatinine-based equation was used as the independent variable. The presented relationship was drug CL versus eCL_CR_, as determined by the Cockcroft-Gault equation. This estimate of CL_CR_, which describes renal function, is still a primary method used in drug labels and in clinical practice for renal dose adjustment, and it is the independent variable used in prior studies of cefepime.

Dosage adjustments of cefepime and taniborbactam will need to be further examined using pharmacokinetic data from patient studies, but it appears from these data that similar recommendations can be made with coadministration. Dosage adjustments are recommended in the cefepime prescribing information for CL_CR_ ≤60 mL/min to compensate for decreases in cefepime CL (5). For CL_CR_ between 30 mL/min and 60 mL/min, the cefepime dosing frequency is recommended to be reduced. Decreases in both the cefepime dosing frequency and the dose are recommended for CL_CR_ <30 mL/min, with the adjustments being dependent upon the CL_CR_ level and the prescribed maintenance schedule. Similar adjustments for patients with renal impairment will be examined in data derived from phase 3 studies for cefepime-taniborbactam to minimize the risk of excessive exposure of both drugs, while still ensuring that pharmacokinetic/pharmacodynamic efficacy targets are met.

Cefepime and taniborbactam are dialyzable, with similar amounts removed during hemodialysis in this study. The amount of cefepime removed during a 3 h hemodialysis session has been reported to be approximately 68%, compared with the 47% found in this study during a 4 h session (5, 13, 16). These amounts may vary due to the system and flow rates used for hemodialysis. For patients undergoing hemodialysis, the dosages and timing of coadministered cefepime and taniborbactam with respect to dialysis will need to be taken into consideration. Also, given the degree of dialyzability of both cefepime and taniborbactam and the likelihood of use in critically ill patients with acute kidney injury, dosage adjustment recommendations will need to be developed for other modalities of renal support.

## MATERIALS AND METHODS

### Study design and subjects.

This was an open-label, single-dose study of the safety, tolerability, and pharmacokinetics of coadministered single doses of taniborbactam and cefepime to subjects with various degrees of renal impairment and matched control subjects with normal renal function. Subjects aged 18 to 80 years were enrolled into 1 of 4 groups based on the level of renal function (renal groups). Renal groups consisted of subjects with normal renal function (“Normal”) with an eCL_CR_ of ≥90 mL/min (Group 1; *n* = 8), subjects with mild renal impairment (“Mild”) with an eGFR of 60 to 89 mL/min/1.73 m^2^ (Group 2; *n* = 6), subjects with moderate renal impairment (“Moderate”) with an eGFR of 30 to 59 mL/min/1.73 m^2^ (Group 3; *n* = 6), and subjects with severe renal impairment (“Severe”) with an eGFR of <30 mL/min/1.73 m^2^ (Group 4; *n* = 6). Attempts were made in the study to enroll subjects within the Severe group with an eGFR value of <15 mL/min/1.73 m^2^. Subjects in the Normal group were matched to subjects with renal impairment with regard to gender, age (±10 years), and weight (±10 kg). A single healthy subject may have been the match for more than one subject with renal impairment. For all subjects in Groups 1 to 4, a single 2 h IV infusion of 2 g cefepime and 0.5 g taniborbactam was administered on day 1.

In addition to the groups enrolled by renal function to study the effect of renal impairment, a group of subjects with ESRD undergoing chronic intermittent hemodialysis were enrolled into a separate group (Group 5; “Dialysis”) to determine the amounts of drug removed by filtration. Hemodialysis used standard commercial dialyzers and membranes. The dialyzer was either a Fresenius F160 or F180 (Fresenius Medical Care North America, Waltham, MA) with blood flow rates (Q_b_) of between 350 and 450 mL/min, dialysate flow rates (Q_d_) of between 500 and 700 mL/min, and reported urea clearances (CL_UREA_) of approximately 270 mL/min. Subjects in the Dialysis group received the same dose of cefepime-taniborbactam as did the subjects in Groups 1 to 4 in each of 2 treatment periods, with the treatments separated by a washout period of 7 to 14 days. A fixed sequence of dialysis timing with respect to dose was used, where the dose was administered prior to dialysis in the first treatment period (“On-dialysis”; Period 1) and administered following dialysis in the second treatment period (“Off-dialysis”; Period 2). Hemodialysis for the On-dialysis period started approximately 4 h after the SOI of cefepime-taniborbactam. For the Off-dialysis period, subjects had the SOI begin approximately 6 h after the start of dialysis, which was after the dialysis had been completed.

The study was performed at two sites in the United States. The study protocol was reviewed and approved by an Institutional Review Board, and the study was performed in accordance with the ethical principles that have their origin in the Declaration of Helsinki. Study conduct complied with the International Council for Harmonisation Guideline for Good Clinical Practice and with applicable regulatory requirements. All subjects provided written informed consent prior to any study-specific procedures.

### Safety.

Safety was assessed based on the occurrence of adverse events and on the evaluation of laboratory tests (chemistry, hematology, urinalysis, and coagulation), physical examinations, vital signs, and 12-lead ECGs. Safety was followed through 7 days post-dose.

### Pharmacokinetic assessments.

The pharmacokinetics of cefepime and taniborbactam in plasma were assessed over 72 to 96 h, depending on the renal group. Subjects in Groups 1 through 4 had blood samples collected through 96 h after the administration of cefepime-taniborbactam on day 1. The pharmacokinetic time points included: pre-dose (within 30 min before dosing), 0.5, 1, 2, 2.25, 2.5, 2.75, 3, 4, 6, 8, 10, 12, 24, 48, 72, and 96 h after SOI. Subjects in Group 1 were not required to have a collection at 96 h.

Subjects in Group 5 had blood samples collected through 72 h after the administration of cefepime-taniborbactam on day 1 of Period 1 and Period 2. The pharmacokinetic time points included: pre-dose (within 30 min before dosing), 0.5, 1, 2, 2.25, 2.5, 2.75, 3, 4, 6, 8, 10, 12, 24, 48, and 72 h after SOI. Additional blood samples, if not already collected by a defined time point, were collected at 1 h after the start of dialysis and again at the end of dialysis. Samples were not collected from Group 5 subjects at 72 h, should this time point have occurred after the next scheduled dialysis.

Urine was collected to further assess taniborbactam pharmacokinetics. Subjects were instructed to empty their bladders completely before study drug administration and just before the end of each collection interval. Urine from each timed interval was collected in a separate container. The exact start dates, stop dates, times of urine collection, and the weight and volume of each collection were recorded. Urine samples were collected within intervals of 0 to 6 h, 6 to 12 h, 12 to 24 h, 24 to 48 h, 48 to 72 h, and 72 to 96 h following the SOI. Group 1 subjects may not have had a urine collection between 72 to 96 h post-SOI. Group 5 subjects did not have a urine collection between 48 to 72 h post-SOI if this time point occurred after their next scheduled dialysis, nor did these subjects have a urine collection between 72 to 96 h post-SOI. Urine samples were not collected from anuric subjects.

Dialysate samples and hemodialysis-associated arterial/venous (A/V) blood samples were collected from Group 5 subjects during Period 1. Spot dialysate samples were taken immediately before the start of dialysis (blank dialysate fluid) and every 30 min after the start of dialysis. Hemodialysis-associated A/V samples were collected immediately at the start of dialysis and thereafter at 1, 2, 3, and 4 h after the start of hemodialysis.

### Bioanalytical methods.

Blood samples collected for pharmacokinetic analysis in the study were processed and assayed for cefepime and taniborbactam in plasma using validated bioanalytical methods. Human dipotassium ethylenediaminetetraacetic acid plasma samples were processed using protein precipitation prior to assay. Urine samples collected for pharmacokinetic analysis in the study were processed and assayed for only taniborbactam using a validated bioanalytical method. Dialysate samples and hemodialysis-associated A/V samples were assayed for taniborbactam and cefepime using validated bioanalytical methods. Acidified urine and dialysate samples were processed using solid-phase extraction prior to assay.

Validated liquid chromatography-tandem mass spectrometry (LC-MS/MS) methods were used to quantitate the concentrations of cefepime and taniborbactam in plasma. The lower limits of quantitation (LLOQ) for these assays were 100 ng/mL and 5.00 ng/mL for cefepime and taniborbactam, respectively. Validated LC-MS/MS methods (LLOQ = 5.00 ng/mL) were used to quantitate the concentrations of taniborbactam in urine. The validated LC-MS/MS method to assay cefepime and taniborbactam in dialysate had LLOQs of 100 ng/mL and 50.0 ng/mL, respectively.

For cefepime assays, separation was accomplished using a SCX Agilent Zorbax 300 column (Santa Clara, CA) at 40°C and isocratic elution using 35 mM ammonium formate as mobile phase A and acetonitrile as mobile phase B. For the taniborbactam assays, separation was accomplished using a Waters Acquity HSS T3 column (Milford, MA) at 50°C and gradient elution using 10 mM ammonium formate with 1% formic acid as mobile phase A and 50:50:1 acetonitrile:methanol:formic acid vol/vol/vol as mobile phase B. All cefepime and taniborbactam assays used a triple quadrupole mass spectrometer (API Triple Quad 5500, AB Sciex, Framingham, MA) equipped with a turbo-ion spray set that was used for detection in positive ion mode, and quantification was based on multiple reaction monitoring. The internal standards used in the cefepime and taniborbactam assays were d_3_-cefepime sulfate and d_4_-taniborbactam, respectively.

Assay accuracy and precision were demonstrated for all assays in the validations, which also included the testing of any required dilutions used in the analysis of study samples. Demonstrated sample stability met the requirements of the sample storage used in the study.

### Pharmacokinetic and statistical analyses.

Individual subject plasma cefepime and taniborbactam pharmacokinetic parameters and individual subject urine taniborbactam pharmacokinetic parameters were calculated using NCA methods (Phoenix WinNonlin, Certara, Princeton, NJ). Estimated plasma pharmacokinetic parameters included the *Cmax*, time to *Cmax* (T_max_), AUC through the last measurable observed concentration (AUC_t_), AUC_inf_, *t*_1/2_, CL, and *V*_Z_. For taniborbactam, using the urine observations, the amount excreted unchanged in urine (Ae), fraction excreted unchanged in urine as the percentage of administered dose (Fe), renal clearance (CL_R_), and nonrenal clearance (CL_NR_) were calculated. The actual sample times were used to calculate the pharmacokinetic parameters. Calculation of the terminal elimination rate (λ_Z_) was based on the best fit of at least 3 concentrations in the observed terminal elimination phase (excluding *Cmax*) and required the goodness of fit statistic (R^2^) to be greater than or equal to 0.80 to be considered acceptable. If a good estimate of λ_Z_ could not be determined for a concentration-time profile, then none of the pharmacokinetic parameters dependent on λ_Z_ were calculated.

The NCA pharmacokinetic parameters were summarized by group using descriptive statistics. Linear regression was used to model changes in pharmacokinetic parameters versus continuous independent variables representative of renal function. Assessed pharmacokinetic parameters included cefepime and taniborbactam *Cmax*, AUC_inf_, CL, *V*_Z_, and *t*_1/2_. Additionally for taniborbactam, CL_R_ was also examined.

For Dialysis subjects (Group 5), the effect of hemodialysis was examined by comparing differences within each subject (On-Dialysis versus Off-Dialysis). The CL_d_ was calculated by taking the individual averaged arterial to venous concentration extraction ratio (CER), multiplying by the reported Q_b_, and correcting for hematocrit (hct). The hct was estimated as 0.47, an assumption based on the fact that all subjects in the group were male. The amounts of drug removed by hemodialysis (A_dial_) were calculated using the area under the excretion rate curves, where the rates were determined using the spot dialysis concentrations and estimated dialysate volumes over a 1 min period using the Q_d_. The fraction of drug removed during hemodialysis, or HER, was then determined by dividing A_dial_ by dose and was expressed as a percentage.
